# Potential of Graphene Nanoparticles for Reducing Cadmium Toxicity and Environmental Contamination

**DOI:** 10.1155/jt/7838711

**Published:** 2026-02-15

**Authors:** Alireza Ghassemi Toussi, Elham Einafshar, Sadaf Sadat Rafati

**Affiliations:** ^1^ Medical Toxicology Research Center, Faculty of Medicine, Mashhad University of Medical Sciences, Mashhad, Razavi Khorasan, Iran, mums.ac.ir; ^2^ Pharmacological Research Center of Medicinal Plants, Mashhad University of Medical Sciences, Mashhad, Razavi Khorasan, Iran, mums.ac.ir; ^3^ Department of Pharmacology, Faculty of Medicine, Mashhad University of Medical Sciences, Mashhad, Razavi Khorasan, Iran, mums.ac.ir

**Keywords:** cadmium, environmental, graphene nanoparticles, nanotechnology, toxicity

## Abstract

This narrative review synthesizes peer‐reviewed studies retrieved from major scientific databases addressing the environmental and biological applications of graphene‐based nanomaterials for cadmium mitigation. Cadmium (Cd) is a nonessential and highly toxic heavy metal with a prolonged biological half‐life and extensive environmental persistence, posing substantial risks to the ecosystem and human health. Exposure to cadmium is linked to nephrotoxicity, carcinogenicity, endocrine disruption, and reproductive issues. This review critically evaluates the emerging role of graphene‐based nanoparticles, particularly graphene oxide (GO) and its functional derivatives, in reducing Cd bioavailability in soil, wastewater, and along the food chain. GO‐based composites can achieve adsorption capacities above approximately 50–500 mg cadmium per gram of material, depending on functionalization and matrix, highlighting their substantial removal potential. Recent evidence demonstrates graphene’s dual functionality: as a potent adsorbent for cadmium ions and as an enhancer of biological tolerance to cadmium‐induced toxicity in both plants and human cells. To our knowledge, this review is the first to comprehensively integrate cross‐disciplinary evidence on (i) the integration of engineered graphene membranes for selective filtration under pressure, (ii) GO‐enhanced soil amendments that reduce cadmium uptake, (iii) thiol‐functionalized composites for precise metal adsorption, and (iv) magnetic graphene nanoparticles enabling suitable cadmium removal. By integrating cross‐disciplinary evidence, we provide a comprehensive roadmap for implementing graphene nanoenabled environmental interventions. Despite promising outcomes, most available evidence is based on laboratory‐scale studies, with limited field‐scale validation and an incomplete understanding of the long‐term environmental fate and ecotoxicity of graphene nanomaterials.

## 1. Introduction

Heavy metals are among the most persistent environmental pollutants, entering the environment primarily through improper disposal of municipal sewage and industrial wastewater [1, 2]. In contrast to organic pollutants, heavy metals are not susceptible to natural decomposition [3, 4]. Their high stability leads to bioaccumulation in the food chain, which poses a threat to the health of plants and animals that consume these foods [5]. Exposure causes chronic and, in some cases, acute toxicity in living organisms, particularly in humans, with complications extending from neurological, cardiovascular, and renal damage to hematological and carcinogenic outcomes [6, 7].

Metal contaminants profoundly impact both soil and water quality. Soil functions not only as a suitable medium for plant growth or a conduit for the entry and exit of pollutants but also as a vector for the dissemination of pollutants to groundwater, the atmosphere, and living organisms [8]. Adsorption of heavy metals onto soil components, including organic matter, clay minerals, and iron/manganese oxides, with mechanisms such as cation exchange, specific adsorption, excretion, and precipitation, governs their fate [9, 10].

Industrial activities are recognized as the primary contributors to heavy metal pollution, with electroplating, battery manufacturing, and electronics production being of particular significance. Many wastewater streams lack treatment systems, discharging notable metal loads into water resources [11]. Treated sludge disposal could be taken up by plants, entering the food cycle and posing risks to ecological systems and human health [12]. While some heavy metals are essential for plant and animal life, many are hazardous even at low concentrations [13]. Metals such as lead, mercury, zinc, nickel, chromium, and cadmium exemplify the range of toxic effects [14]. Cadmium, in particular, stands out for its high toxicity. It can move through certain soil conditions, enter the food chain, and pose threats to the health of plants, animals, and humans [15]. Its solubility and bioavailability in soil are dependent on soil properties, including texture, pH, organic matter, and cation exchange capacity (CEC) [16]. In plants, exposure to cadmium can impair photosynthesis and nutrient balance, leading to a reduction in crop quality [17]. Cadmium accumulation in aquatic environments affects the survival of fish, invertebrates, and algae, disrupting the ecosystem’s structure and function [18]. Eventually, ingestion of cadmium‐contaminated water, seafood, or crops by humans is linked to a spectrum of health complications, underscoring the urgent need for novel and effective remediation strategies across various environmental compartments [19].

Nanostructured materials, particularly graphene and its derivatives, offer attractive properties for the remediation of heavy metals in different matrices, including soil, wastewater, and plants [20, 21]. These special features include a large surface area [22], tunable porosity [23], controllable surface functional groups (e.g., amine, carboxyl, and hydroxyl groups), and magnetic separability [24]. Thus, the administration of graphene nanomaterials as a cadmium remediation agent could potentially reduce its environmental burden and exposure risk.

The present study assesses graphene‐based materials and derivatives for cadmium remediation. We aimed to determine: (i) the effectiveness across different matrices (soil, water, and plant systems) and human cells, (ii) comparative performance among graphene‐related materials (e.g., graphene oxide, reduced graphene oxide, functionalized graphene, and graphene‐based composites), and (iii) ecotoxicity and environmental safety implications. It is important to note that related 2D materials, such as graphitic carbon nitride (g‐C3N4) nanosheets, are not counted among the primary graphene‐related scope, unless a specific subsection explicitly addresses them only as comparators. This review was conducted through a comprehensive search of previous research conducted by the authors and a thorough review of the literature from reliable scientific sources.

## 2. Cadmium

### 2.1. Physical and Chemical Properties of Cadmium

Cadmium is a chemical element with the symbol Cd and atomic number 48. This metal is characterized by its soft and lustrous appearance, as well as its white hue. Its chemical properties are similar to those of zinc and mercury metals, often considered analogous [25]. Like zinc, cadmium typically exhibits an oxidation state of +2 in various compounds [26]. The average cadmium concentration in the Earth’s crust ranges from 0.1 to 0.5 parts per million (ppm). Cadmium is a constituent of most minerals and is a byproduct of zinc production [27]. Historically, cadmium has been used as a corrosion‐resistant steel coating [28]. Cadmium compounds are utilized as red, orange, and yellow pigments to stabilize the colors of glass and plastics [29]. However, cadmium’s toxicity has led to its limited utilization, with nickel–cadmium batteries being replaced by nickel–metal hydride and lithium‐ion batteries in many applications [30].

### 2.2. The Environmental Effects of Cadmium

The environment comprises various components, including soil, which receives industrial and agricultural waste and pollutants. The presence of pollutants in soil and water has been demonstrated to directly correlate with adverse health effects in plants and humans. Heavy metals’ nondegradability and capacity to elicit physiological effects in living organisms underscore the potential for even low concentrations of these elements to induce groundwater and soil contamination, thereby jeopardizing human health and environmental quality [31]. The rate of cadmium methylation is significantly lower in comparison to that of elements such as mercury, arsenic, and lead. Only two bacterial species, *Pseudomonas* sp. and *Staphylococcus aureus,* have been observed to be capable of methylating cadmium in water environments [32]. Due to its greatest toxicity to plants compared to other heavy metals, cadmium should receive more attention [33]. The primary sources contributing to cadmium distribution in the environment include fossil fuels, waste disposal in plastic bags, insecticides, sewage sludge in agriculture, and phosphate fertilizers [34]. The most salient symptoms of cadmium toxicity in plants include the cessation of root growth, reduced chlorophyll synthesis, and a concomitant decrease in vegetable quality [35]. Furthermore, acute toxicity resulting from cadmium exposure has been demonstrated to be lethal to animals and birds and to cause severe poisoning in aquatic animals [36].

### 2.3. Occupational Exposure to Cadmium Poisoning

The most hazardous occupations and practices that can lead to cadmium poisoning include working in battery manufacturing plants, welding and soldering jobs, smelting and mining, working in the textile industry, using cadmium alloys or paints and plastics containing this element, working in the jewelry industry, working in stained glass, and working in the recycling industry [37]. Additionally, individuals engaged in composting and waste collection may also be exposed to dust containing cadmium [38]. Another potential source of exposure to this toxic metal is the burning of municipal waste [39]. However, research has demonstrated that various sources, including everyday activities, can lead to cadmium exposure. For instance, cigarette smoke has been found to contain cadmium [40]. Additionally, cultivating crops such as potatoes and vegetables in soils with elevated cadmium levels can contaminate them [41].

### 2.4. Clinical Manifestations of Cadmium Poisoning

Cadmium is primarily absorbed through inhalation and ingestion [42]. Following absorption, it is transported throughout the body and is predominantly bound to metallothionein, a sulfhydryl group‐containing protein that can protect against heavy metal toxicity and other oxidative stresses [14]. Cadmium accumulation in the human body is predominantly observed in the kidneys and liver. Notably, there is an absence of a specific excretory mechanism for cadmium within the body [33].

Cadmium, a toxic heavy metal, has been linked to a variety of diseases, including chronic kidney disease, osteomalacia, acute heart failure (dilated cardiomyopathy), secondary hypertension, and atherosclerosis [43]. The extent of cadmium’s effect on the body is directly related to the volume of this element, the amount of this element that has entered the body, the length of time the person has been exposed to the component, and the level of the body’s response to this element [33]. Cadmium enters the body through the respiratory system. Thus, the absorption of cadmium from the lungs is more efficient than from the intestine, with 50% of the cadmium inhaled through cigarette smoke being absorbed. Consequently, smokers exhibit average concentrations of cadmium in their blood that are 4–5 times higher than those observed in nonsmokers, and concentrations in their kidneys are typically 2–3 times higher [44]. Inhalation of substantial quantities of cadmium in a brief period can result in symptoms similar to those of the flu, including fever, chills, and muscle aches. In the long term, cadmium exposure can result in lung damage, shortness of breath, chest pain, cough, and, in severe cases, death [45, 46]. While research has demonstrated that cadmium may impact various physiological systems by inducing lipid oxidation in cells and disrupting the antioxidant system, the precise mechanism by which cadmium affects the cardiovascular system remains to be elucidated [46, 47]. However, higher blood cadmium levels have been associated with increased mortality from coronary heart disease. Furthermore, cadmium exposure has been linked to the prevalence and inflammation of carotid plaques, which are accumulations of lipids and other substances in the arteries of the neck, and an elevated risk of ischemic stroke, characterized by an interruption in blood supply to the brain [47]. The toxic effects of cadmium in humans also include the kidneys. Cadmium has been observed to accumulate in the proximal tubules, resulting in the manifestation of Fanconi syndrome. Furthermore, exposure to cadmium has been demonstrated to heighten the risk of developing chronic kidney disease [48]. A body of research has demonstrated a strong correlation between urinary excretion of cadmium and its accumulation in the human body. Consequently, urinary cadmium excretion serves as a marker for the body’s cadmium burden and can be utilized to predict mortality in patients with heart failure and acute myocardial infarction [49]. Cadmium has also been shown to directly inhibit calcium absorption from the intestine and disrupt collagen metabolism, leading to osteomalacia and osteoporosis [50]. Cadmium has been demonstrated to diminish the body’s immune system, thereby increasing susceptibility to bacterial and viral infections, and consequently, the risk of developing cancer and mortality [51].

## 3. Graphene Compounds

The term “graphene” is derived from the prefix “graph,” which is derived from the term “graphite,” and the suffix “‐ene,” which is derived from the C‐C double bonds [52]. Graphene is a two‐dimensional nanoparticle of carbon material that is arranged in a hexagonal lattice. Essentially, it is a sheet of carbon with four bonds [53]. Nanographene synthesis involves a process known as “dehydrogenation,” which involves the selective removal of hydrogen atoms from organic carbon and hydrogen molecules [54]. The properties of graphene include transparency, thermal and electrical conductivity, impermeability, stiffness, and tensile strength resistance [46, 55, 56]. It absorbs only 2%–3% of reflected light, resulting in unique optical properties and high transparency [57]. It is also noteworthy that graphene exhibits a thermal conductivity that is double that of diamond and 13 times that of copper [58]. The unique atomic arrangement of carbon atoms in graphene allows its electrons to move easily at very high speeds without much scattering, and it is used as an electrical conductor [59]. The high permeability of graphene ensures that even helium, a particularly small atom, cannot readily traverse a graphene layer. Consequently, a layer of atoms can act as an excellent barrier to gases and liquids [60]. The pores in graphene act selectively, allowing the material to regulate the permeability of gases [61]. Despite its relatively brittle nature, graphene can exhibit up to 25% flexibility and elasticity, rendering it highly advantageous for applications in flexible electronics [62]. Moreover, the potential of graphene in biomedicine is significant due to its large surface area and electron mobility, which have led to its use in thermal ablation of highly resistant cancer cells, electronic interfaces with living cells and neural tissue, luminescent graphic labels for bioimaging and drug delivery, and cancer treatment [63–66]. In addition, studies have demonstrated that graphene oxide nanosheets exhibit a substantial inhibitory effect on the growth of *E. coli* bacteria. The bactericidal action of graphene oxide nanosheets is attributed to the damage they cause to bacterial cell membranes [67]. The application of graphene layers in hygiene products and food packaging is driven by their ability to maintain product freshness and extend shelf life [68]. While pristine graphene exhibits many advantageous properties, it has a high tendency to aggregate and sediment in aqueous environments, low compatibility with different matrices, and limited interaction sites for contaminant adsorption. To address these limitations, functionalized graphene derivatives and composites substantially improve dispersibility, stability, and adsorption capacity, enabling optimal performance across diverse applications [69].

The term “graphene derivatives” is used to describe a variety of substances, including graphite, nanographene platelets, and graphene oxides. Graphite, a form of carbon, is created by arranging individual graphene sheets in a layered structure [70]. Nanographene platelets, another type of graphene derivative, are composed of one or more graphene layers with a thickness ranging from 0.34 to 100 nm [71]. Typically, these platelets are formed by stacking two graphene layers on a silicon dioxide substrate [72]. The advantages of graphene oxide (GO) include excellent mechanical, thermal, and electrical properties, extensive chemical bonding with various resins, and the ability to filter out harmful organic and inorganic substances [73]. GO is a monoatomic layered material and an oxidized form of graphene to which oxygen‐containing functional groups (hydroxyl, epoxy, and carboxyl groups) have been added. Unlike graphene, which is hydrophobic, GO exhibits hydrophilic properties and disperses in aqueous solutions. The presence of oxygen groups facilitates enhanced interaction with other materials and allows the formation of covalent bonds between GO and other materials [74, 75].

Finally, these nanoparticles represent a subject of significant interest within the domain of technology, primarily due to their distinctive characteristics, which encompass mechanical, thermal, electrical, and optical properties [76]. According to previous research, graphene nanoparticles have been used in electrical equipment, batteries, and capacitors as energy storage, as well as in the environment as a sensor and a nanomaterial for removing air and water pollutants [77, 78]. Despite promising results, gaps remain in their long‐term stability, potential ecotoxicity, and scalable production. In this study, we conducted a review of various studies conducted in the field of removing metal pollutants, including cadmium, from biological environments (polluted water and soil) by graphene nanoparticles. We investigated their properties in improving the growth and biological reproduction of plants and animals in these polluted environments.

## 4. The Effect of Graphene Nanoparticles on Reducing Cadmium Contamination

The contamination of water resources with toxic pollutants, including heavy metals and organic compounds, represents a significant environmental challenge in the context of the industrial age. These pollutants, which have their origins in sources such as industrial, agricultural, and domestic waste, have the potential to harm aquatic life and render water unsafe for human consumption. In light of these considerations, the removal of pollutants from wastewater represents a long‐standing and persistent challenge in environmental management. Conventional remediation methods include precipitation, oxidation, ion exchange, and coagulation–flocculation. However, most of these approaches are limited by substantial maintenance and operational costs, the generation of hazardous sludge, and reduced sustainability, ultimately diminishing their long‐term applicability and overall efficiency. One promising alternative is adsorption technology, offering a cost‐effective, expedient, and efficient physicochemical strategy for removing organic pollutants and heavy metals. In recent years, researchers have investigated the potential of functionalized graphene‐based composite materials for the purification of contaminated water. These materials exhibit a substantial surface area and high levels of reactivity, along with distinctive physicochemical characteristics that render them highly promising adsorbents. These materials, which can be produced using techniques such as chemical vapor deposition and chemical exfoliation, have been demonstrated to be effective in the removal of heavy metals and organic pollutants from water. The appropriate dosage of adsorbent is a crucial element in the treatment of industrial wastewater, as it can significantly impact the cost‐effectiveness of the process. As evidenced by research, augmenting the dosage of adsorbent results in enhanced pollutant removal due to an expanded surface area, thereby facilitating a greater number of adsorption sites. However, an excess of adsorbent can result in aggregation, which in turn reduces the number of available adsorption sites. Furthermore, alterations in the pH of the solution may occur [46, 79–81].

Graphene, when modified or used in composites, has become a prominent material in the field of adsorption technology. Its application in this domain facilitates the identification and elimination of heavy metals, such as cadmium, which has an acceptable limit of 0.1 mg/L and can induce a range of physiological complications if released into the environment. Graphene‐based adsorbents have been demonstrated to exhibit superior performance compared to other carbon adsorbents, including activated carbon [82].

### 4.1. Soil

The study conducted by Rassaei et al. aimed to investigate the potential of GO as a modifier of heavy metals in soil, as well as its influence on the growth of corn plants. Furthermore, this study examined the impact of heavy metal cadmium and GO on soil pH, biomass production, and cadmium accumulation in corn plants. The study’s findings indicated that both cadmium and GO exert a considerable influence on soil pH, with GO exhibiting a beneficial impact and Cd exerting an adverse effect. The findings revealed that GO mitigates the detrimental impact of cadmium on biomass and functions as a chelating agent to diminish cadmium toxicity and enhance nutrient uptake in corn plants. The study demonstrated that GO resulted in a notable enhancement in chlorophyll content in corn plants, whereas cadmium accumulated in roots and shoots, leading to a reduction in plant growth and biomass. Ultimately, the study concluded that GO can be employed as a soil amendment to mitigate the detrimental effects of cadmium on soil and plant health (Table 1, entry 1) [83].

**Table 1:** Effects of graphene nanoparticles on the absorption and reduction of cadmium toxicity in soil samples.

Soil	Nanoparticle	Nanoparticle effects	Reference
	GO	↓ Cd toxicity.	[83]
		↑ Nutrient uptake in corn plants.	
		↑ Chlorophyll content in corn plants.	
		↑ Plant growth and biomass.	

	ZnO/GO	↓ Soil Cd content.	[84]
		↑ Soil pH.	
		↑ Soil cation exchange capacity.	
		↓ Time‐dependent cadmium toxicity.	

	Reduced carbon dioxide (PRGO)	↑ Cd absorption at pH > 4.	[85]
		The maximum Cd absorption capacity was 434.78 mg/g.	
		Selective absorption of copper, zinc, lead, and Cd from soil.	
		Prevention of absorption of potassium, sodium, and magnesium salts from soil.	
		Maintaining natural soil texture for plant growth.	

*Note:* Cd = cadmium, ZnO/GO = zinc oxide/graphene oxide, ↑ = increase or enhancement, ↓ = decrease or inhibition.

Abbreviation: GO = graphene oxide.

In a recent study, Li et al. explored the potential of zinc oxide/graphene oxide (ZnO/GO) nanocomposite as a promising novel amendment for the remediation of cadmium‐contaminated soil. The findings demonstrated that a single‐day ZnO/GO treatment was efficacious in reducing the cadmium content of soil and increasing the pH and CEC in soil through the interaction between ZnO/GO and soil organic acids. Moreover, this study employed the use of the nematode *Caenorhabditis elegans* (*C. elegans*) as a living organism to assess the safety of the soil and the impact of ZnO/GO on cadmium‐contaminated soil. The findings demonstrated that ZnO/GO induced cadmium excretion and reduced cadmium storage in *C. elegans* by increasing the expression of the ttm‐1 gene and decreasing the expression of the cdf‐2 gene, which are responsible for the transport and accumulation of cadmium, respectively. Furthermore, it was determined that the toxicity and ecological characteristics of cadmium‐contaminated soil exhibited time‐dependent improvement in response to ZnO/GO (Table 1, entry 2) [84].

In the study conducted by Xu et al., the impact of porous nanomaterials comprising reduced carbon graphene oxide (PRGO) on the adsorption and reduction of cadmium metal from soil was examined. The findings of this study indicate that PRGO contains a significant quantity of oxygen‐containing functional groups, namely, carboxyl and hydroxyl groups, which enhance the efficiency of cadmium adsorption at pH values exceeding 4. The maximum capacity for cadmium adsorption, as reported, was 434.78 mg/g of material. Furthermore, PRGO exhibits selective adsorption of copper, zinc, lead, and cadmium while preventing the adsorption of potassium, sodium, and magnesium salts from the soil solution. Consequently, the natural soil texture conducive to plant growth remains intact (Table 1, entry 3) [85].

A review of the existing literature reveals a consensus that GO‐based materials often mitigate cadmium toxicity in soil environments and improve the physicochemical properties of the soil, thereby enhancing the absorption of mineral elements required for plant growth. Potential mechanisms include adsorption, altered cadmium bioavailability, and indirect effects on soil biota. However, further research is needed to determine the long‐term fate of graphene compounds in soil and the relationship between adsorption metrics and ecological risk.

### 4.2. Wastewater

In a separate study conducted in 2024, graphene nanosheets modified with hydrogen and hydroxyl groups were examined for their potential in filtering and removing heavy metal ions from contaminated water sources. The findings indicated that the pores of graphene nanosheets modified with hydrogen or hydroxyl groups possess the selective capacity to filter heavy metal ions from contaminated water, thereby enhancing the purification process. Additionally, this study examined the impact of varying nanosheet pore sizes and applied pressures. The findings indicated that at low pressures, the functional groups under examination, particularly –OH, exert a considerable influence on the interaction between water molecules and the membrane, thereby facilitating the purification process. At elevated pressures, the pore size is instrumental in the purification and removal of heavy metals. Ultimately, an evaluation of the findings revealed that pore size, optimization of the applied pressure, and the type of functional group significantly enhance the efficiency of graphene membranes in separating heavy metals [86].

In 2019, Jafaryan et al. conducted a study to investigate the effect of graphene nanoparticles on the removal of cadmium ions from contaminated aqueous solutions. The findings indicated that graphene nanoparticles exhibited a cadmium removal efficiency of 70% (502 mg/g) under optimal conditions. The most optimal conditions for cadmium adsorption were identified as pH 6.18, temperature 25°C, graphene nanoparticles at a dose of 1 g/L, and ambient cadmium content of 970 mg/L. Additionally, this study employed a magnetic surfactant to facilitate the recovery and reuse of graphene nanoparticles, thereby enabling the formation of noncovalently functionalized magnetically modified graphene. In the course of the magnetic recovery of the graphene nanoparticles, a combined effort was made to successfully recover both the graphene and magnetic surfactant components. This was achieved by adjusting the surface charges through a deliberate alteration of the pH of the solution. The removal behavior of the graphene nanoparticles was studied using an HNO_3_ solution, and it was found that the recovered nanoparticles could be reused (Table 2, entry 1) [87].

**Table 2:** Effects of graphene nanoparticles on the absorption and reduction of cadmium toxicity in wastewater samples.

Wastewater	Nanoparticle	Nanoparticle effects	Adsorption capacity/removal efficiency	Reference
	Graphene nanoparticle	Cd removal efficiency was 70% (502 mg/g). The best conditions for Cd adsorption were a temperature of 25°C and graphene nanoparticles at a dose of 1 g/L. The possibility of recovering nanoparticles using magnetic surfactants and separating them from each other by changing the pH of the solution.	502 mg/g (70%)	[87]

	GO + Thiol functional group (GO‐SOxR)	↑ The adsorption capacity of high‐metal Cd with the thiol group. Maximum Cd absorption capacity was 208 mg/g.	208 mg/g	[88]

	GO + FeCl_3_ + 3‐mercaptopropyl (M‐Fe_3_O_4_‐GO)	The maximum Cd adsorption capacity was 125 mg/g. Complete recovery of M‐Fe_3_O_4_‐GO adsorbent using an external magnetic field from aqueous solution.	125 mg/g	[89]

	GO + magnetic ethylenediamine (MDFGO)	The maximum adsorption capacity for Cd was 99.4 ± 0.6%.	99.4 ± 0.6%	[90]

	GO	↑ The metal adsorption under the influence of pH and ionic strength. The maximum adsorption capacity of cadmium at pH = 6.0 ± 0.1 and *T* = 303 K has been reported to be approximately 106.3 mg/g.	106.3 mg/g	[91]

	GO	↑ The Cd removal efficiency in a dose‐dependent manner from 6.29% to 96.72%. ↑ The Cd adsorption with increasing solution pH from 2.02 to 4.01. The maximum Cd adsorption capacity was 44.64 mg/g.	44.64 mg/g	[92]

	Graphene nanoparticle + ferroferric oxide (Fe_3_O_4_–GS)	The maximum adsorption capacity for Cd was 27.83 mg/g. Metal adsorption occurs mainly through ion exchange.	27.83 mg/g	[93]

	3D‐sulfonated reduced graphene oxide (3D‐SRGO)	The maximum Cd adsorption capacity was reported to be 234.8 mg/g at pH = 6.0. The cation exchange process is an important mechanism for Cd removal by 3D‐SRGO. HNO_3_ solution at pH = 0.3 can effectively remove 97.23% of Cd from 3D‐SRGO, thereby facilitating the recovery of nanoparticles.	234.8 mg/g	[94]

	GO + Poly‐2‐aminothiophenol (PATP@GO)	The maximum adsorption capacity of PATP@GO nanocomposite for Cd (125.0 mg/g) at pH = 7 was reported to be 87.3%.	125.0 mg/g (87.3%)	[95]

	GO + thiacalix[4]arenetetrasulfonate (TCAS‐rGO)	The adsorption of Cd on TCAS‐rGO was significantly affected by pH. ↑ Selective adsorption of Cd at neutral pH by TCAS‐rGO. The maximum adsorption capacity of the adsorbent was 128 mg/g for Cd.	128 mg/g	[96]

	Carboxymethyl cellulose–graphene oxide nanocomposite (CMC–GO)	The optimum pH, adsorbent dosage, time, and removal percentage were 5.5, 1.5 g/L, 60 min, and 82.5% for Cd. The maximum adsorption capacity for Cd was 134.04 mg/g.	134.04 mg/g	[97]

	Amine‐functionalized graphene oxide (GO‐NH_2_)	The maximum adsorption capacity for Cd is 10.04 mg/g.	10.04 mg/g	[98]

	Magnetic graphene oxide nanocomposite + diethylenetriamine agent (GO–Fe_3_O_4_–DETA)	The nanocomposite allowed the detection of 0.40 μg/L of Cd. The maximum Cd adsorption capacity was 59.88 mg/g.	59.88 mg/g	[99]

	GO	The maximum adsorption capacity was determined to be 23.9 mg/g in the pH range of 6–7. The dominant mechanisms in Cd adsorption are electrostatic forces and cation exchange between Cd and oxygen‐containing functional groups.	23.9 mg/g	[100]

	GO	The maximum Cd adsorption capacity was 1.779 mmol/g. ↑ Cd adsorption with increasing pH from 2 to 6.	1.779 mmol/g	[101]

	Graphene nanosheets + amino functional groups (amino/triamino)	The lowest detectable concentration of Cd in contaminated environments was 5 ng/L. The maximum adsorption capacity of GO with one amine functional group was 179 mg/g for Cd. The maximum adsorption capacity of GO with three amine functional groups was determined to be 236 mg/g for Cd.	179 mg/g and 236 mg/g with one and three amine functional groups, respectively.	[102]

	Hydrolyzed polyacrylonitrile (hPAN) + GO + 4‐vinylpyridinium (PVPI)	The maximum adsorption capacity for Cd was 6.6% (0.05 mg/g).	6.6%	[103]

	Magnetic graphene nanocomposite + 5‐aminoisophthalic acid (mGOAIPA)	The highest adsorption capacity for Cd ions was (120.51 mg/g) at pH = 6.5.	120.51 mg/g	[104]

	Graphene carbon nitride composite + ruthenium + cobalt oxide (Ru@Co_3_O_4_@g‐C_3_N_4_)	The maximum adsorption capacity of Cd ions was 564.5 mg/g. The removal mechanism was based on the formation of a stable covalent bond between the unpaired electrons of the triazine ring and the functional groups.	564.5 mg/g	[105]

	rGO + Halophilic bacterium Halomonas BVR 1	The maximum adsorption capacity was reported to be 20.30 mg/g for lead. The purification of wastewater samples was found to be 98% efficient. The regeneration of nanoparticles with hydrochloric acid was accomplished with relative ease.	20.30 mg/g	[106]

	Graphene oxide/magnetic cellulose + β‐cyclodextrin	β‐cyclodextrin–GO has a maximum adsorption capacity of 303.98 mg/g.	303.98 mg/g	[107]

	GO + Cysteine‐rich metal‐binding protein (MBP) and Cyanobacterium metallothionein (SmtA)	The Cd adsorption capacity of the nanoparticle composite with SmtA was 7.70 mg/g, which was higher than the value observed for GO nanosheets without SmtA (2.34 mg/g).	7.70 mg/g	[108]

	GO + *α*, β, and γ cyclodextrins (CDs–GO)	β‐CD–GO exhibited optimal removal characteristics at a pH = 7, with an efficiency of Cd removal of 90%. Both γ‐CD–GO and α‐CD–GO exhibited high adsorption capacity for Cd (222.22 mg/g).	222.22 mg/g	[109]

*Note:* Cd = cadmium, ↑ = increase or enhancement.

Abbreviation: GO = graphene oxide.

In a separate study, Pirveysian and Ghiaci prepared GO nanoparticles with a thiol functional group (organic sulfur compound) (GO‐SOxR) using sodium sulfide. The presence of immobilized dense thiol groups on the surface of thiol‐functionalized GO results in a high‐metal adsorption capacity. In this study, the use of a coating with TiO_2_ or SiO_2_ was also employed to enhance the efficiency of the adsorbent. The removal of heavy metal ions, including cadmium, nickel, lead, and zinc, from aqueous solutions was investigated using GO‐SOxR@TiO_2_ and GO‐SOxR@SiO_2_ composites. Furthermore, the adsorption of heavy metal ions by GO‐SOxR, GO‐SOxR@TiO_2_, and GO‐SOxR@SiO_2_ was compared. Ultimately, the findings indicated that the adsorption capacity of GO was markedly enhanced by sulfur functionalization and TiO_2_ coating, reaching an adsorption capacity of 208 mg/g (Table 2, entry 2) [88].

In a separate study, Liu et al. synthesized graphite oxide using FeCl_3_, resulting in a magnetic graphene composite. Additionally, in this study, the thiol groups of (3‐mercaptopropyl)trimethoxysilane (MPTES) were employed to enhance the performance of the synthesized nanoparticles (M‐Fe_3_O_4_‐GO). The maximum adsorption capacity of M‐Fe_3_O_4_‐GO for cadmium, as determined from the Langmuir isotherm, was found to be 125 mg/g. Moreover, the M‐Fe_3_O_4_‐GO adsorbent exhibited an extraordinary adsorption capacity for cadmium, which was attributed to the synergistic interactions between thiol groups, Fe_3_O_4_ nanoparticles, and graphene nanosheets. Additionally, the M‐Fe_3_O_4_‐GO adsorbent exhibited the capacity for complete separation from the contaminated aqueous solution under study through the utilization of an external magnetic field, thereby enabling its reuse (Table 2, entry 3) [89].

In a study conducted by Ghorbani et al., a new adsorbent, namely, magnetic ethylenediamine‐functionalized graphene oxide (MDFGO), was synthesized and subsequently employed for the removal of heavy metal pollutants, including cadmium and lead, from wastewater samples of battery and electroplating origin. A series of experiments was conducted to determine the optimal conditions for the effective adsorption of lead (II) and cadmium (II) under controlled circumstances. The parameters investigated encompassed pH level, adsorbent dosage, shaking speed, and adsorption time. The outcomes of these experiments indicated that the parameters that resulted in the most effective adsorption at a given time were found to be 6.2 mmHg, 0.33 mg/kg, 500 rpm, and 11 min, respectively. Ultimately, the MDFGO adsorbent demonstrated the effective removal of lead (II) and cadmium (II) to a degree of 99.6 ± 0.5% and 99.4 ± 0.6%, respectively (Table 2, entry 4) [90].

In the study conducted by Zhao et al., the impact of GO nanosheets comprising multiple layers of graphite on the adsorption and removal of cobalt and cadmium ions from aqueous solutions contaminated with heavy metals was examined. An investigation was conducted to ascertain the impact of pH, ionic strength, and humic acid on the adsorption of these metals. The findings of the study indicated that the adsorption of the examined metals on GO nanosheets is markedly influenced by pH and ionic strength. Additionally, the functional groups with a high oxygen content on the surfaces of GO nanosheets played a significant role in the adsorption process. The presence of humic acid was observed to reduce the adsorption of cadmium and cobalt on GO nanosheets at pH values below 8. The maximum adsorption capacity of cadmium and cobalt on GO nanosheets at a pH of 6.0 ± 0.1 and a temperature of 303 K was reported to be approximately 106.3 and 68.2 mg/g, respectively (Table 2, entry 5) [91].

In the study conducted by Xue et al., GO nanosheets were synthesized and employed for the adsorption of cadmium in acidic aqueous solutions. The findings indicated that the removal efficiency of cadmium increased in a dose‐dependent manner (from 0.02 to 2.00 g/L) when GO nanoparticles were used. The efficiency increased from 6.29% to 96.72%. Nevertheless, the removal efficiency remained unaltered with an escalation in the quantity of the adsorbent. Furthermore, the adsorption of cadmium was found to increase significantly with an increase in solution pH from 2.02 to 4.01. The maximum cadmium adsorption capacity was approximately 44.64 mg/g, which was achieved with an adsorbent dosage of 0.50 g/L. Ultimately, the study demonstrated that the efficiency of cadmium removal increased with an increase in the dosage of the adsorbent and the initial pH of the solution. Additionally, ion exchange, electrostatic adsorption, and physical adsorption contribute to the adsorption mechanisms of GO nanosheets (Table 2, entry 6) [92].

Ahmad et al. investigated the impact of GO resin in conjunction with polyethylenimine (PS@GOA) on the adsorption and concentration of heavy metals, specifically cadmium and lead, from wastewater samples. The findings demonstrated that the PS@GOA adsorbent can be utilized repeatedly for up to 72 cycles without a reduction in extraction efficiency. The recovery of analytes exhibited a range of 98.0%–104%. The prepared adsorbent exhibits chemical stability, with no evidence of GO leaching from the separation column. Furthermore, the presence of hydrophilic functional groups and an abundance of affinity enhance the capacity for metal adsorption from aqueous environments. Furthermore, this study revealed that the optimal pH for the highest efficiency of nanoparticles in the removal and concentration of lead (II) and cadmium (II) was 6 and 8, respectively [110].

In a study conducted by Li et al. in 2022, the impact of MgAl double‐layer hydroxide nanosheets on GO (GL) and its magnetic product (Fe_3_O_4_@GL) was examined as potential adsorbents for heavy metals, including lead, cadmium, and copper. The findings of this study indicate that the nanoparticles under examination, which possess functional groups, facilitate the formation of extensive surface areas conducive to the adsorption of heavy metals. Furthermore, GL and Fe_3_O_4_@GL demonstrate optimal heavy metal removal under neutral and weak alkalinity conditions. The maximum adsorption capacity of GL for lead, cadmium, and copper was determined to be 226.98, 76.67, and 89.26 mg/g, respectively. Furthermore, the adsorption capacity of Fe_3_O_4_@GL for lead, cadmium, and copper was also reported to be 213.96, 70.26, and 80.72 mg/g, respectively. The primary metal removal mechanisms include surface complexation of metal hydroxyl groups and metal carbonates with divalent metal cations [111].

In a related study conducted by Guo et al., the adsorption capabilities of magnetic graphene nanoparticles in conjunction with ferroferric oxide (Fe_3_O_4_–graphene sulfonate [GS]) were demonstrated, showcasing their effectiveness in removing lead, chromium, cadmium, nickel, and mercury from aqueous solutions. The study demonstrated that Fe_3_O_4_–GS could be readily recovered from the water by a magnetic current within 1 minute. The metal adsorption capacities were found to be 22.07, 17.29, 27.95, 23.03, and 27.83 mg/g for nickel (II), chromium (VI), lead (II), mercury (II), and cadmium (II), respectively. Additionally, it was determined that the adsorption of metals occurred primarily through ion exchange (Table 2, entry 7) [93].

In the study conducted by Wu et al., it was discovered that three‐dimensional sulfonated reduced graphene oxide (3D‐SRGO) nanoparticles possess a porous structure, a large surface area, hydrophilic properties, and a high adsorption capacity for the removal of cadmium (II) ions from wastewater. The results demonstrated that the adsorption of cadmium was initially rapid, followed by a slower adsorption process, with approximately 93% of the adsorption occurring within 20 min. The maximum adsorption capacity of the investigated nanoparticles was reported to be 234.8 mg per gram at a pH of 6.0. Thermodynamic parameters demonstrated that the adsorption reaction of cadmium (II) ions on 3D‐SRGO is a spontaneous process. The findings of X‐ray photoelectron spectroscopy (XPS) and Fourier transform infrared spectroscopy (FT‐IR) analysis suggest that the cation exchange process plays a substantial role in the removal of cadmium by 3D‐SRGO. Regeneration experiments demonstrated that an HNO_3_ solution at pH = 0.3 could effectively remove 97.23% of cadmium from 3D‐SRGO, thereby facilitating the recovery of the nanoparticles (Table 2, entry 8) [94].

In the study conducted by Akhdhar et al., the efficacy of a combined approach involving poly‐2‐aminothiophenol (PATP) and GO (PATP@GO) was assessed for the removal of heavy metals, specifically cadmium and lead, from aquatic environments. The incorporation of PATP was observed to enhance the adsorption efficiency of GO, with a notable increase from 61.3% to 98.6% for lead and from 34.6% to 87.3% for cadmium. Furthermore, the findings demonstrated that the PATP@GO nanocomposite manifested an exceptional efficiency and adsorption capacity, with a reported value of 98.6% for lead (142.9 mg/g) at a pH of 5%, and 87.3% for cadmium (125.0 mg/g) at a pH of 7 (Table 2, entry 9) [95].

The present study, conducted by Liu, sought to examine the impact of reduced graphene oxide (rGO) with a thiacalix[4]arenetetrasulfonate–functionalized reduced graphene oxide (TCAS‐rGO) on its capacity as an adsorbent for the removal of lead (II) and cadmium (II) ions from aquatic solutions. The adsorption of these metals on TCAS‐rGO was investigated as a function of pH, contact time, metal ion concentration, and temperature, and it was found that the adsorption of lead (II) and cadmium (II) on TCAS‐rGO was significantly influenced by pH. At a neutral pH, TCAS‐rGO exhibited a high degree of selective adsorption for lead (II) and cadmium (II) in comparison to other metals, including sodium (I), potassium (I), magnesium (II), and strontium (II). Furthermore, the adsorption capacity of TCAS‐rGO demonstrated an increase with rising pH levels. The maximum adsorption capacity of the adsorbent was determined to be 230 mg/g for lead (II) and 128 mg/g for cadmium (II). Moreover, the study demonstrated the effectiveness of TCAS‐rGO as an adsorbent, with the material exhibiting no degradation after four adsorption/desorption cycles (Table 2, entry 10) [96].

In a recent study, Pan et al. examined the impact of the white fungus *Phanerochaete chrysosporium* (WRF) on the biodegradation of the antibiotic sulfamethoxazole in an aqueous medium containing the heavy metal cadmium. In this study, the effect of rGO on the activity of the fungus in biodegradation and the removal of environmental stress caused by cadmium was also investigated. The findings indicated that WRF can biologically remove the antibiotic sulfamethoxazole from aqueous media containing 16 mg/L of cadmium, with a removal efficiency ranging from 55.6% to 28.6%. Additionally, the use of GO nanoparticles has been demonstrated to enhance the efficacy of the fungus in question for the removal of sulfamethoxazole. The nanoparticles effectively safeguard the mycelium structure of WRF against the oxidative stress of cadmium, while simultaneously enhancing the production of manganese peroxidase in WRF [112].

In a recent study, Shakil et al. examined the potential of carboxymethyl cellulose–graphene oxide nanocomposite (CMC–GO) as an adsorbent for the removal of lead and cadmium from aqueous solutions. The results demonstrated that the optimal pH, adsorbent dosage, time, and removal percentage were 5.0, 1.0 g/L, 60 min, and 96.3% for lead and 5.5, 1.5 g/L, 60 min, and 82.5% for cadmium. The maximum adsorption capacity for cadmium and lead was reported to be 134.04 and 213.21 mg/g, respectively (Table 2, entry 11) [97].

Song et al. (2019) investigated the impact of porous graphene oxide–polypyrrole (pGO/PPy) polymer on the electrochemical detection of cadmium (II). The findings demonstrated that 1–100 μg/L of 3D GO with a porous structure exhibited high linear sensitivity in the detection of cadmium in the environment, capable of detecting 0.05 μg/L of cadmium (II) in the environment, which is considerably lower than the cadmium limit set by the World Health Organization (WHO) [113].

In a separate study, Huang et al. synthesized amine‐functionalized graphene oxide (GO‐NH_2_). In this study, 3‐aminopropyl triethoxysilane was employed for the synthesis of nanoparticles. The findings demonstrated that GO‐NH_2_ was capable of effectively adsorbing and removing copper, lead, cadmium, and chromium ions, with maximum adsorption capacities of 26.25, 71.89, 10.04, and 280.11 mg/g, respectively. Consequently, it can be employed in the remediation of industrial wastewater contaminated with heavy metals (Table 2, entry 12) [98].

The adsorption and elimination of cadmium and lead ions from aquatic and vegetable environments were investigated using a magnetic GO nanocomposite with a diethylenetriamine agent (GO–Fe_3_O_4_–DETA). The synthesis of this nanocomposite entailed the formation of amide bonds between the amine groups of diethylenetriamine and the oxygen‐containing functional groups (epoxy and carboxyl) of GO. The findings of the study indicated that the investigated nanocomposite resulted in the detection of 0.38 and 0.40 μg/L of lead and cadmium, respectively. Furthermore, the maximum adsorption capacity for lead and cadmium is 172.41 and 59.88 mg/g, respectively. It should be noted that the recovery rate differed when the method was applied to real samples (Table 2, entry 13) [99].

In a separate study, Bian et al. examined the impact of GO with oxygen‐containing functional groups on the adsorption of cadmium ions. The findings of this study demonstrated that the presence of oxygen‐containing functional groups on GO surfaces enhanced the adsorption of cadmium. The maximum adsorption capacity was determined to be 23.9 mg/g within the pH range of 6–7. The results of the FT‐IR and XPS analysis, conducted before and after the adsorption of cadmium on GO, indicated that the dominant mechanisms responsible for cadmium adsorption were electrostatic forces and cation exchange between cadmium and the functional groups with oxygen on GO (Table 2, entry 14) [100].

In a study published in 2016, Hemidouche and colleagues investigated the adsorption and removal effects of GO nanoparticles. The findings of this study indicate that GO nanoparticles are effective in removing lead and cadmium cations from contaminated aqueous solutions. The maximum adsorption capacity for lead and cadmium was reported to be 0.946 and 1.779 mmol ions/g, respectively. Furthermore, the study indicated that an increase in pH value results in a negative surface charge on the nanoparticles, thereby enhancing the adsorption of lead and cadmium cations. Consequently, an increase in pH from 2 to 6 resulted in a notable enhancement in cadmium adsorption, from 0.195 to 0.509 mmol/g (Table 2, entry 15) [101].

Sayar et al. investigated the impact of graphene nanosheets with amino functional groups (amino/triamino) on the adsorption of lead and cadmium ions. The findings indicated that the lowest detectable concentrations of lead and cadmium in contaminated settings were 0.9 μg/L and 5 ng/L, respectively. The maximum adsorption capacity of GO with a single amino functional group was found to be 359 mg/g for lead and 179 mg/g for cadmium. Furthermore, the maximum adsorption capacity of GO with three amino functional groups was determined to be 461 and 236 mg/g for lead and cadmium, respectively (Table 2, entry 16) [102].

Shen and Chen (2015) conducted a comparative analysis of the efficacy of GS nanosorbent, rGO, and GO in the adsorption of aquatic pollutants. The GS nanosorbent was prepared via the synthesis of sulfanilic acid. The findings demonstrated that this compound was capable of surmounting the constraints associated with GO, thereby enhancing its hydrophilicity and aggregation. The sulfonic acid utilized also demonstrated a robust capacity to adsorb positively charged pollutants, including cadmium. The maximum adsorption capacity of GS for phenanthrene, methylene blue (MB), and cadmium was reported to be 400, 900, and 58 mg/g, respectively, which was significantly higher than the values observed for rGO and GO [114].

In a recent study, Neves et al. examined the efficacy of polymer nanocomposite membranes comprising hydrolyzed polyacrylonitrile (hPAN), GO, and 4‐vinylpyridinium (PVPI) in removing toxic metals from industrial wastewater. The findings of the study indicated that the hydrophilicity, morphology of the layers, and oxygenated functional groups (OH and COOH groups) enhanced the efficiency of the nanocomposite membrane. The efficiency of the hPAN membrane with three PVPI/GO layers in the removal of metal ions was demonstrated to be superior to that of other polymer nanocomposite membranes. The maximum adsorption capacity for metal ions cadmium, nickel, copper, chromium, and lead was 6.6% (0.05 mg/g), 23.1% (0.45 mg/g), 50.4% (0.83 mg/g), 64.3% (3.14 mg/g), and 99.9% (4.08 mg/g), respectively (Table 2, entry 17) [103].

In 2024, Lellala et al. employed magnetite nanoparticles (Fe_3_O_4_) integrated onto the surfaces of GO sheets to desulfurize and eliminate heavy metals from industrial wastewater. The results demonstrated that the studied nanoparticles were capable of removing heavy metals (lead, cadmium, copper, manganese, and chromium) and sulfur with an efficiency of over 97%. The mechanism of adsorption for the removal of heavy metals was reported to include electron acceptor–donor interactions, ion exchange, and electrostatic interactions, in addition to surface complexation, which demonstrated excellent hybrid synergy [115].

In the study conducted by Zhou et al., the impact of montmorillonite‐reduced graphene oxide (M‐rGO) nanoparticles on the elimination of heavy metals, including cadmium and cationic dyes (e.g., MB), from industrial wastewater was examined. The results indicated that the functional groups comprising oxygen and carbon were found to be instrumental in the adsorption of both cadmium and MB. Additionally, the hydrogen bonds and hydrophobicity between M‐rGO and MB played a pivotal role in the adsorption process, which encompassed both chemical and physical adsorption. To achieve the most effective enhancement of the dynamic adsorption capacity of cadmium and MB on M‐rGO, it is necessary to reduce the initial concentration of the pollutant, increase the pH value, and slow the pumping speed of the contaminated solution [116].

In a recent study, Alazzawi et al. fabricated three‐dimensional graphene sheets from date sap, which is rich in carbon, and then combined them with silver nanoparticles. The present study was conducted to investigate the properties of the nanocomposite, its capacity to remove heavy metal pollutants from wastewater, and its antibacterial properties. The findings indicated that date sap graphene nanosheets are capable of removing heavy metals, including cadmium, lead, zinc, and chromium, from water with removal efficiencies of 68%, 39%, 10%, and 27%, respectively. Furthermore, the incorporation of silver nanoparticles enhanced the removal efficiency, with the removal efficiency of zinc, chromium, and cadmium reported to be 22%, 33%, and 75%, respectively. Moreover, the antibacterial capacity of graphene was enhanced by up to 100% following its modification with silver nanoparticles [117].

In a recent study, Del Rosso et al. examined the sensor properties of GO nanoparticles in conjunction with gold for the detection of heavy metals in aqueous environments. The findings indicated that the graphene–gold nanoparticles (Gr/Au) exhibited a high thermodynamic affinity for mercury (*K* = 3.1 × 10^7^ L mol^−1^) and, to a lesser extent, for cadmium (8.5 × 10^6^ L mol^−1^) and lead (1.1 × 10^7^ L mol^−1^). Consequently, this study demonstrated that the detection of heavy metals in contaminated environments with the assistance of graphene gold nanoparticles enables the removal of these metals from said environments [118].

In a related study conducted by Ismardi et al., it was demonstrated that a zinc oxide nanocomposite comprising a polyvinyl alcohol functional group and graphene (ZnO–PVA–Graphene) is capable of detecting cadmium in a concentration range of 0–80 ppm, with a detection limit of 9.88 ppm. Additionally, it was indicated that the optimal quantity of graphene in the synthesized composite for the detection of cadmium is 1.5 wt% [119].

In 2024, Nakhlestani et al. investigated the performance of a 5‐amino isophthalic acid‐modified magnetic graphene nanocomposite (mGOAIPA) for the removal of lead and cadmium metal ions from aqueous media. The findings indicated that the mGOAIPA nanocomposite exhibited the highest adsorption capacity for cadmium ions (120.51 mg/g) and lead ions (198.79 mg/g) at a pH of 6.5. Therefore, this study demonstrated that magnetic graphene oxide functionalized with organic polymers represents an effective approach for the removal of heavy metal pollution, particularly lead and cadmium ions, from aqueous media (Table 2, entry 18) [104].

In 2024, Elamin et al. synthesized the Ru@Co_3_O_4_@g‐C_3_N_4_ nanocomposite, a graphene carbon nitride composite comprising ruthenium and cobalt oxide, and investigated its efficacy in removing heavy metals, specifically copper and cadmium, from polluted waters. The findings of the study indicated that the maximum adsorption capacity of copper and cadmium ions by the Ru@Co_3_O_4_@g‐C_3_N_4_ nanocomposite was 696.9 and 564.5 mg/g, respectively. Additionally, the mechanism by which the studied nanocomposite removes the ions under investigation was reported to be based on the formation of a stable covalent bond between the unpaired electrons of the triazine ring and functional groups (Table 2, entry 19) [105].

In the study conducted by Rajesh, the impact of the halophilic bacterium Halomonas BVR 1 in conjunction with rGO was examined as an adsorbent for the removal of metal ions, specifically lead, cadmium, and zinc, from aqueous solutions. The maximum adsorption capacity was reported to be 73.58, 20.30, and 42.53 mg/g for lead, cadmium, and zinc, respectively. Moreover, the purification of wastewater samples was found to be 98% efficient. The regeneration of nanoparticles with hydrochloric acid was accomplished with relative ease. Ultimately, the study demonstrated that the microbially modified rGO biosorbent exhibits a notable capacity for the separation of these metal ions in a real wastewater sample (Table 2, entry 20) [106].

In a study published in 2020, Zhang and colleagues examined the efficacy of various graphene compounds, specifically graphene (G), amine‐modified graphene (GNH), and GO, in removing metal pollutants, including cadmium, from the aquatic environment of the algae *Scenedesmus obliquus*. The results demonstrated that the impact of graphene compounds on cadmium toxicity was in the order GO > *G* > GNH. The discrepancy in the impact of these compounds on cadmium toxicity may be attributed to the distinct surface oxygen‐containing functional groups present in these nanomaterials [120].

In a further study, Rahman and Raheem explored the impact of graphene oxide/magnetic cellulose, modified with β‐cyclodextrin (CN/IGO/Cel), on the elimination of cadmium ions. The findings demonstrated that the incorporation of β‐cyclodextrin into the IGO/Cel composite enhanced the removal efficacy of cadmium ions and the adsorption capacity. The maximum adsorption capacity of the investigated nanoparticle was determined to be 303.98 mg/g for cadmium ions (Table 2, entry 21) [107].

Yang et al. (2012) synthesized GO nanosheets in conjunction with a cysteine‐rich metal‐binding protein (MBP) and cyanobacterium metallothionein (SmtA). The composite exhibited markedly high selectivity for cadmium adsorption, with SmtA demonstrating a binding and adsorption capacity of 7.70 mg/g of cadmium, a significantly higher value than that observed for GO nanosheets lacking SmtA (2.34 mg/g) (Table 2, entry 22) [108].

In the research study conducted by Einafshar et al., the impact of graphene oxide nanocomposites conjugated with α, β, and γ cyclodextrins (CDs–GO) on the elimination of cadmium ions from polluted water was examined. This study investigated the adsorption capacity of cadmium ions, and the impact of various experimental parameters, including contact time, initial metal ion concentration, and initial pH, on the adsorption process. The findings demonstrated that the optimal removal characteristics of cadmium were exhibited by β‐CD–GO at a pH of 7, with an efficiency of cadmium removal of 90%. It was observed that the adsorption of cadmium ions by γ‐CD–GO occurred at a rate of more than 50% and that equilibrium was attained within 2 h.

The adsorption capacity of cadmium for both γ‐CD–GO and α‐CD–GO was found to be 222.22 and 208.33 mg/g, respectively, suggesting a significant difference between the two (Table 2, entry 23) [109].

Collectively, the reviewed investigations consistently emphasize the high cadmium absorption capacity of functionalized graphene‐based nanomaterials across aqueous environments. Notably, composites like thiol‐functionalized GO and magnetic graphene materials show improved selectivity and biocompatibility, highlighting their suitability for targeted remediation. However, variations in study setups, such as adsorbent dosage, temperature, and pH, limit cross‐study comparisons. Furthermore, the effects of functionalization on microbial communities and ecotoxicity are context‐dependent. Mechanistic patterns involve competition with native ions, surface complexation, and possible redox interactions. Although laboratory results are considerable, there is a lack of field‐scale validation and assessment of long‐term environmental behavior.

### 4.3. Plants

In 2020, Li et al. investigated the effects of GO nanosheets on the uptake and plant toxicity of the heavy metal cadmium. In this study, the impact of cadmium at a concentration of 1 mg/L and GO nanosheets at concentrations of 0, 1, and 10 mg/L on the growth, germination of rice seeds, and the uptake and accumulation of cadmium in the roots and shoots of the plant were evaluated. The findings indicated that GO nanosheets, due to their hydrophilic characteristics, can absorb and transport water in a concentration‐dependent manner, thereby enhancing rice seedlings’ germination and subsequent growth. Furthermore, they mitigated the deleterious effects of cadmium on rice seed germination. Furthermore, GO nanosheets at a concentration of 10 mg/L were observed to enhance cadmium uptake by both the rice plant’s roots and shoots. The present findings suggest that GO may have the capacity to modify the impact of cadmium on the germination of rice seeds, as well as its uptake and accumulation in the roots and shoots of rice seedlings (Table 3, entry 1) [121].

**Table 3:** Effects of graphene nanoparticles on the absorption and reduction of cadmium toxicity in plants.

Plants	Nanoparticle	Plants	Nanoparticle effects	Reference
	GO	Rice (*Oryza sativa* L.)	↓ The detrimental effects of Cd on rice seed germination. ↑ Germination. ↑ Growth of rice seedlings.	[121]

	Graphitic carbon nitride nanosheets (g‐C_3_N_4_ NSs)	Soybean (*Glycine max* L.)	↓ Cd toxicity. ↑ Plant growth. ↓ Oxidative damage. ↑ Pectin content and modifying its demethylation led to changes in root cell wall components and retention of Cd in root cells up to 82.8%. ↓ Cd influx to plant stems. ↑ Photosynthetic efficiency by increasing chlorophyll content.	[122]

	Graphene + C_3_N_4_ nanosheets	Soybean (*Glycine max* L.)	↑ 49.5% and 63.4% in dry weight of soybean shoots and roots, respectively. ↓ Oxidative stress. ↑ Photosynthesis and beard activity. ↑ Soil nitrogen cycling, nodulation, and nodule nitrogen fixation capacity.	[123]

	GO + rhizobium (Rh)	Soybean (*Glycine max* L.)	↑ Plant root growth. ↑ Antioxidant enzyme activity. ↑ Soybean yield and sustainable growth potential in alkaline soil conditions.	[124]

	GO	Lettuce	↓ Cd toxicity. ↓ Reactive oxygen species and malondialdehyde content. ↑ Photosynthesis, stomatal conductance, chlorophyll content, and ribulose‐1, 5‐bisphosphate carboxylase. ↑ Oxygenase concentration in plant cells.	[125]

	GO	Duckweed	↑ Cd accumulation in plants. ↑ The phytoremediation efficiency of duckweed.	[126]

	GO	*Lonicera japonica*	↑ Cd absorption and accumulation in plant roots and aerial parts. ↑ Dry weight of root and aerial part biomass, photosynthesis rate, carbon content, and oxygen release per unit leaf area. ↓ Cd toxicity. ↑ Phytoremediation capability.	[127]

	GO	Rice (*Oryza sativa* L.)	↓ Cd toxicity. ↑ Cd uptake by plant roots and its accumulation. ↑ Seed germination and seedling growth.	[128]

*Note:* Cd = cadmium, ↑ = increase or enhancement, ↓ = decrease or inhibition.

Abbreviation: GO = graphene oxide.

In the study conducted by Xu et al. in 2024, the impact of graphitic carbon nitride nanosheets (g‐C_3_N_4_ NSs) on the reduction of the toxicity of the metal pollutant cadmium in soybean (*Glycine max* L.) was investigated. The application of g‐C_3_N_4_ NSs resulted in a notable enhancement in plant growth and a reduction in oxidative damage. Additionally, g‐C_3_N_4_ NSs regulated the components of the root cell wall by increasing the pectin content and modifying its demethylation through the increased activity of pectin methylesterase, thus increasing the retention of cadmium in root cells by 82.8% and reducing its translocation to the plant stem. Furthermore, the efficiency of photosynthesis was enhanced by an increase in chlorophyll and sugar content. In conclusion, the findings of this study indicate that g‐C_3_N_4_ NSs have the potential to significantly reduce cadmium toxicity in soybean plants (Table 3, entry 2) [122].

In a separate study, Xu examined the impact of g‐C_3_N_4_ nanosheets on the growth parameters of *Glycine max* L. As in the preceding study, the incorporation of g‐C_3_N_4_ NSs into soil resulted in a 49.5% and 63.4% increase in the dry weight of soybean shoots and roots, respectively. It mitigated oxidative stress and enhanced photosynthesis and root activity. Furthermore, the cadmium content was reduced from 35.7% to 54.1%. Furthermore, the application of g‐C_3_N_4_ NSs in cadmium‐contaminated soil enhanced soil nitrogen cycling, nodulation, and nodule nitrogen fixation capacity, resulting in a 12.4% and 43.2% increase in shoot and root nitrogen content, respectively (Table 3, entry 3) [123].

In a parallel study, the impact of GO nanoparticles in conjunction with Rhizobium (Rh) on the expression of growth and yield genes in soybean (*Glycine max* L.) in alkaline soil environments was examined. The findings of this study indicated that the GO + Rh treatment resulted in enhanced plant root growth. Additionally, the activity of antioxidant enzymes was observed to increase significantly in comparison to the control group. Ultimately, the findings indicated that the combination of GO and Rh enhanced soybean yield and sustained growth potential in alkaline soil conditions (Table 3, entry 4) [124].

In a separate study conducted by Gao et al., the potential impact of GO sprays on photosynthesis and antioxidant systems in lettuce plants subjected to cadmium stress was examined. The findings indicated that the application of 30 mg/L GO resulted in a notable reduction in the manifestations of cadmium toxicity in lettuce. Moreover, it diminished the accumulation of reactive oxygen species, malondialdehyde content, and antioxidant enzyme activities. Furthermore, the treatment enhanced photosynthesis, stomatal conductance, chlorophyll content, ribulose‐1, 5‐bisphosphate carboxylase, and oxygenase concentration in the plant cells (Table 3, entry 5) [125].

The effective remediation of heavy metal pollution in aquatic environments using phytoremediation, facilitated by the use of aquatic plants such as duckweed, represents a viable solution. In the study conducted by Yang et al., the impact of GO on the accumulation of cadmium in duckweed was examined. The findings indicated that GO enhanced the accumulation of cadmium in duckweed. Additionally, following a six‐hour treatment of duckweed with cadmium, the results demonstrated that the cadmium content in the leaves was 1.4 times and in the roots was 1.25 times that of the control group, while the cadmium content in the aquatic environment was 0.67 times that of the control group. Consequently, the transfer of cadmium to the duckweed plant was markedly enhanced by the influence of GO. The combination of GO and cadmium was observed under an electron microscope to adhere to the cell surface of duckweed in the form of white crystals. Ultimately, the study concluded that GO can enhance the phytoremediation efficacy of duckweed in aquatic environments subjected to cadmium‐induced stress (Table 3, entry 6) [126].

In a recent study, Liu et al. explored the potential of GO in conjunction with *Lonicera japonica* for the bioremediation of heavy metal cadmium in a plant culture medium. In this study, treatments of 0, 10, 50, and 100 mg/L of GO and varying concentrations of cadmium (0, 5, and 25 mg/L) were employed. The findings demonstrated that GO at the lowest concentration examined (10 mg/L) was capable of further stimulating *L. japonica* to absorb and accumulate cadmium at a concentration of 5 mg/L in roots and shoots. Furthermore, the research observed an enhancement in the dry weight of root and shoot biomass, as well as an augmentation in the photosynthesis rate, the carbon content, and the oxygen release per unit leaf area in the subject plant. Consequently, the findings indicated that GO markedly diminished the detrimental effects of cadmium on *L. japonica*, thereby enhancing its phytoremediation capabilities (Table 3, entry 7) [127].

In a study conducted by Yin et al., the effects of GO and cadmium on seed germination, growth, and cadmium uptake by rice (*Oryza sativa*) in a cadmium‐contaminated aqueous medium were investigated. The findings indicated that GO can rapidly absorb cadmium from solution, such that an increase in the concentration of GO results in a decrease in the residual cadmium concentration. Additionally, the findings indicated that the rate of seed germination, root growth, and shoot length exhibited a decline with an increase in cadmium concentration. The inhibitory effects can be mitigated by the presence of GO. The presence of GO has been observed to enhance the uptake of cadmium by plant roots, leading to increased accumulation within the plant. However, with increasing GO concentration (exceeding 500 mg/L), the uptake rate increased, resulting in accumulation within the plant stem. In conclusion, it is evident from the findings of this study that GO can modify the impact of cadmium on the processes of seed germination, seedling growth, and cadmium uptake from contaminated solutions via its adsorption (Table 3, entry 8) [128].

In a study conducted by Mitra et al., it was also demonstrated that a 3D reduced GO electrode can be employed for the detection and bioaccumulation of cadmium within bacterial cells (*Klebsiella michiganensis*) and rice tissues. Several studies have demonstrated that the use of a cadmium‐resistant plant growth promoter (PGPR) rhizobacterial strain, namely, *Klebsiella michiganensis*, can facilitate the bioremediation and removal of cadmium metal pollutants. As a consequence of its ability to absorb and remove cadmium from natural environments, this strain has been observed to enhance plant growth. The findings of the study indicated that the combination of graphene nanoparticles with the rhizobacterium in question enhances the bioremediation capacity for the removal of cadmium from the environment. This results in increased rice plant growth [129].

The results of existing evidence demonstrated the capacity of graphene‐containing nanoparticles to reduce heavy metals, including cadmium, from plant culture media. Some plant studies suggest that GO and other graphene‐based materials have promising adsorption efficiency in plants, showing potential for integration into soil amendments and filtration systems. The reduction in cadmium toxicity engenders an increase in growth factors within plants, thereby elevating the quality of agricultural produce. On the other hand, several studies indicate that graphene‐based materials increase cadmium uptake, which is desirable in phytoremediation contexts. However, from a food safety perspective, the GO‐enhanced cadmium absorption by edible crops is undesirable.

### 4.4. Food and Drink

In a related investigation, the impact of magnetic GO nanosheets functionalized with cysteine on the removal of cadmium was examined. The study demonstrated that cysteine exhibits a higher affinity for cadmium adsorption. Consequently, it can be concluded that GO nanosheets modified with cysteine can be used as effective adsorbents for heavy metals and the quantitative determination of cadmium in a range of food samples, including milk, wheat, shrimp, and rice. The optimal pH for cadmium removal was reported to be within the range of 5–9, with a level of 24.39–30.30 mg/g (Table 4, entry 1) [130].

**Table 4:** Effects of graphene nanoparticles on the absorption and reduction of cadmium toxicity in food and drink.

Food and drink	Nanoparticle	Food and drink	Nanoparticle effects	Reference
	GO + cysteine‐functionalized	Milk, wheat, shrimp, and rice	↑ Cd absorption.	[130]
			↓ Cd toxicity.	
			The optimal pH for cadmium removal was in the range of 5–9, with a level of 24.39–30.30 mg/g.	

	GO + azophenol groups	Barley, rice, milk, and fish	The detection limit of Cd was 0.4 μg/L.	[131]
			The maximum Cd adsorption capacity in the studied foods was 54.94–73.5 mg/g.	
			Adsorption increased with increasing pH to 7.	

	Graphene + dipyridylamine group	Seafood	The maximum Cd adsorption capacity was 392 mg/g.	[132]

*Note:* Cd = cadmium, ↑ = increase or enhancement, ↓ = decrease or inhibition.

Abbreviation: GO = graphene oxide.

In a 2015 study by Moghadam Zadeha et al., the impact of GO nanosheets in conjunction with dithizone on the adsorption and removal of lead and cadmium from contaminated foods was investigated. The findings indicated that the incorporation of dithizone enhanced the thermal and chemical stability of graphene nanosheets. Furthermore, the adsorption efficiency of heavy metal contaminants in foodstuffs, particularly fruits, which are more prone to contamination due to their high water content, was enhanced. The maximum adsorption capacity for cadmium and lead was determined to be 254 and 474 mg, respectively [133].

In 2018, Sa’adi and Es’haghi functionalized magnetic GO nanosheets using azophenol groups. The compound was employed for the separation of cadmium metal from a variety of foodstuffs, including barley, rice, milk, and fish. The results demonstrated that the detection limit of cadmium in the studied environment was 0.4 μg/L. Additionally, the maximum cadmium adsorption capacity in the studied foods was reported to be 54.94–73.5 mg/g. The study of the effect of pH also demonstrated that an increase in pH to 7 enhances the efficiency of cadmium adsorption. Nevertheless, a pH value exceeding 7 results in the precipitation of cadmium ions in the environment, thereby reducing their adsorption and removal efficiency (Table 4, entry 2) [131].

In the study conducted by Karimi et al., the impact of graphene nanosheets modified with a dipyridylamine group on the adsorption of cadmium ions in seafood samples was examined. The findings indicated that the maximum adsorption capacity for cadmium was 392 mg per gram. Additionally, in this study, various acidic solutions, including HCl, HNO_3_, HClO_4_, and H_2_SO_4_, as well as a mixture of these with thiourea, were employed for the removal of ions from graphene nanosheets. The findings indicated that the optimal detergent solution was 8 mL of 0.1 mol/L thiourea in conjunction with 0.1 mol/L HCl (Table 4, entry 3) [132].

Studies have indicated that the utilization of graphene nanoparticles has the potential to mitigate the toxicity of the metal pollutant cadmium in food sources, particularly seafood. This mitigation is hypothesized to result in an enhancement of the food chain. However, further research is necessary to substantiate this application and to exclude the possibility of graphene‐induced toxicity in food. Moreover, mechanistic links between surface chemistry, cadmium binding, and bioavailability in edible tissues require further study.

### 4.5. Human Cells

In a study published in 2020, Abbasabadi and colleagues investigated the efficacy of nanomagnetic composites of GO functionalized with naphthalene‐1‐thiol and Fe_3_O_4_ (Fe_3_O_4_@NpSH‐GO, NMNT‐GO) in removing cadmium from blood samples. This finding indicates that the adsorptive capacity of Fe_3_O_4_@NpSH‐GO and GO in blood samples at pH 6.0 is 185.3 and 33.7 mg/g of cadmium, respectively (Table 5, entry 1) [134].

**Table 5:** Effects of graphene nanoparticles on the absorption and reduction of cadmium toxicity in human cell lines.

Human cells	Nanoparticle	Cell line	Nanoparticle effects	Reference
	GO + Naphthalene‐1‐thiol + Fe_3_O_4_ (Fe_3_O_4_@NpSH‐GO, NMNT‐GO)	Blood samples	The adsorption capacity of GO and Fe_3_O_4_@NpSH‐GO was 33.7 and 185.3 mg/g of Cd in blood samples at pH = 6.0, respectively.	[134]

	rGO	HepG2	rGO has no harmful effect on HepG2 cells. ↓ Cd toxicity. ↑ Cell viability. ↓ Cd‐induced cell cycle arrest by rGO. ↑ Antioxidants.	[135]

	ZnO/GO nanocomposites	HepG2	↓ Cytotoxicity and genotoxicity of Cd. ↓ Cellular uptake of Cd through competitive inhibition. ↑ Expression of MRP1 and facilitated Cd excretion from cells. ↓ Cd‐induced liver and bile duct cell damage.	[136]

*Note:* Cd = cadmium, ZnO/GO = zinc oxide/graphene oxide, HepG2 = hepatoblastoma cell line, MRP1 = multidrug resistance–associated protein 1, ↑ = increase or enhancement, ↓ = decrease or inhibition.

Abbreviations: GO = graphene oxide, rGO = reduced graphene oxide.

The extensive utilization of rGO for the remediation of cadmium in biological systems has prompted concerns regarding its potential impacts on human health and the environment. Ahamed et al. investigated the combined impact of cadmium and GO nanoparticles on human liver cells (HepG2). The findings of the study indicated that rGO did not exert any detrimental effects on HepG2 cells at concentrations up to 50 μg/mL, whereas cadmium demonstrated dose‐dependent cytotoxicity at concentrations ranging from 1 to 10 μg/mL. Additionally, it was found that a noncytotoxic concentration of rGO (25 μg/mL) could effectively mitigate the toxic effects of cadmium (2 μg/mL). This included decreased cell viability, lactate dehydrogenase release, and alterations in cell morphology. Furthermore, rGO exposure led to a notable reduction in cadmium‐induced cell cycle arrest, caspase (3 and 9) enzyme activity, and loss of mitochondrial membrane potential. In addition, the effects of cadmium on the production of pro‐oxidants (e.g., hydrogen peroxide) and the reduction of antioxidants (e.g., glutathione and superoxide dismutase) could be counteracted by the application of rGO. The observed effects can be attributed to the capacity of rGO to adsorb cadmium on its surface, thereby reducing the uptake of cadmium into cells and its bioavailability in HepG2 cells (Table 5, entry 1) [135].

Cadmium is one of the 20 identified hazardous substances with the capacity to cause significant damage to liver cells. Nevertheless, the synthesis of efficacious liver antidotes with optimal detoxification capacity for cadmium removal represents a significant challenge. In a study conducted by Liu et al., the researchers investigated the potential of ZnO/GO nanocomposites to mitigate cadmium‐induced liver injury. The present study employed two models. The *in vitro* model utilized the human hepatocellular carcinoma (HepG2) cell line, whereas the *in vivo* model employed male mice. The findings of both studies demonstrated that the nanocomposite effectively impeded the cytotoxicity and genetic toxicity of cadmium, exhibiting optimal antagonistic efficacy. Approximately 90% of the nanocomposites were observed to competitively inhibit cellular cadmium uptake, a process that was facilitated by the release of zinc ions and subsequent excretion through the kidney. Furthermore, the nanocomposites under investigation were observed to enhance the expression of multidrug resistance–associated protein 1 (MRP1), which facilitated the excretion of cadmium from the cell. Ultimately, the damage to liver cells and bile ducts induced by cadmium was mitigated following treatment with ZnO/GO, which may be attributed to the enhanced biocompatibility of GO (Table 5, entry 3) [136].

In light of the pervasive presence of biological pollutants and the consequent rise in cancer incidence, the identification of effective mitigation strategies is imperative. Collectively, the studies mentioned earlier indicate that graphene nanoparticles can reduce cadmium toxicity, thus lessening related harmful effects, such as changes in cellular structure, metabolic processes, and modifications to the cell cycle. However, the majority of human cell studies relied on data from cancer‐derived cell lines, such as HepG2, during short‐term exposures, primarily focusing on cell viability and oxidative stress endpoints. To elucidate the full scope of implications of graphene nanoparticles in human health risk, future studies should incorporate primary cells and a broader panel of endpoints.

## 5. Impact of Graphene Nanoparticles on Living Organisms

Several studies have been conducted to investigate the removal of cadmium metal ions by graphene nanoparticles. The findings of these studies indicate that, in addition to their beneficial effects in removing and reducing cadmium ions from the environment, these nanoparticles also cause ecotoxicological effects. Indeed, graphene influences cadmium handling in cells and plants in two ways: First, GO/rGO surfaces bind cadmium, reducing its availability and cellular uptake. Second, graphene alters the expression of metal transport and detoxification genes as well as antioxidant systems. Outcomes depend on graphene type, dosage, exposure conditions, and the organism.

In a research study undertaken by Gao et al. in 2019, the toxicity of cadmium to photosynthesis and oxidative stress in wheat seedlings was investigated in the presence of GO. The findings indicated that GO had minimal impact on the growth of the examined seedlings at low concentrations, whereas concentrations exceeding 20 mg/L resulted in adverse effects. The combined treatment of cadmium–GO demonstrated that GO was toxic at a concentration of 5 mg/L, significantly increasing cadmium accumulation in plant roots through its uptake and reducing plant biomass. Furthermore, in this instance, a plethora of physiological parameters exhibited significant deterioration, including net photosynthetic rate, stomatal conductance, transpiration rate, and maximum photochemical efficiency of photosystem II. Additionally, the photosynthetic electron transfer rate, chlorophyll content, and ribulose‐1,5‐bisphosphate carboxylase/oxygenase were found to be markedly compromised, while the intracellular CO_2_ concentration underwent a substantial increase. Additionally, the researchers indicated that these alterations can be attributed to the mechanisms of toxicity due to oxidative stress (Table 6, entry 1) [137].

**Table 6:** Toxic effects of graphene nanoparticles in addition to their benefits in absorbing and removing cadmium from contaminated environments.

Toxic effects	Nanoparticle	plants	Nanoparticle effects	Reference
	GO	Wheat	↑ Cd accumulation in plant roots. ↓ Plant biomass. ↓ Photosynthesis rate, stomatal conductance, transpiration rate, chlorophyll content, and ribulose‐1, 5‐bisphosphate carboxylase/oxygenase. ↑ Intracellular CO_2_ concentration. ↑ Oxidative stress.	[137]

	GO	Wheat	↓ Root growth is due to the combination of GO and Cd. ↑ Cytochrome P450 activity. ↓ Tubulin content. ↑ Transport and accumulation of cadmium in plant roots and damage to root cell structures. ↓ Efficiency of water and nutrient absorption. ↓ Plant growth and development.	[138]

	GO	Rice (*Oryza sativa* L.)	GO converts the mineral form of Cd into a form that can be absorbed by plants. ↑ Cd content in rice seedlings increased by up to 12.5%. Causes indirect toxicity to plants in contaminated soils.	[139]

	GO	Algae	↑ Destruction of cellular structure and physiological function at high concentrations of GO. ↑ Toxic effect on *M. aeruginosa*. ↑ Cell death. ↑ Levels of reactive oxygen species and malondialdehyde.	[140]

	GO	*Microcystis aeruginosa*	↓ Cellular biomass and photosynthetic parameters. ↓ Photochemical processes and cellular metabolism. ↑ Oxidative stress levels.	[141]

	GO	(Algae) *Chlorella vulgaris*	↑ Cd toxicity. ↑ Expression of genes related to metal transport, including ABCG37 and ZIP4. ↓ Metabolic pathways are involved in synthesizing amino acids, fatty acids, and carbohydrates.	[142]

	GO	*Corchorus olitorius* L.	↓ Plant growth. ↑ Oxidative stress.	[143]

*Note:* Cd = cadmium, CO_2_ = carbon dioxide, ↑ = increase or enhancement, ↓ = decrease or inhibition.

Abbreviation: GO = graphene oxide.

In a related study, Gao and Song examined the impact of GO on the absorption and toxicity of cadmium in wheat seedlings. The findings indicated that in comparison to treatments involving the administration of cadmium or GO individually, the combined treatment of GO and cadmium resulted in a notable reduction in the growth of roots and their associated components. Furthermore, the combined treatment of GO and cadmium increased cytochrome P450 activity and resulted in a reduction in tubulin content. The presence of GO resulted in increased transport and accumulation of cadmium within the plant roots, which subsequently led to damage to cellular structures, including cell membranes and chloroplasts. This resulted in root blockage and a reduction in the efficiency of water and nutrient absorption, which ultimately inhibited wheat growth and development. Ultimately, the findings indicated that GO augmented the phytotoxicity of cadmium by facilitating its absorption and accumulation in the roots of wheat seedlings, thereby reducing the concentration of cadmium in the surrounding environment (Table 6, entry 2) [138].

In a separate study, the impact of GO on the uptake of cadmium from rice soil was examined. The findings indicated that the capacity of the nanoparticles to absorb and remove cadmium from contaminated soil was enhanced by the presence of oxygen‐containing functional groups and an extensive surface area. Additionally, the maximum cadmium absorption capacity was reported to be 265.8 mg/g. Furthermore, the evaluations demonstrated that GO facilitates the conversion of the mineral form of cadmium into a form that can be absorbed by plants, resulting in an increase in the cadmium content in rice seedlings by up to 12.5%. In light of these findings, it can be concluded that although GO is effective in removing heavy metal pollutants, it also causes indirect toxicity to plants in contaminated soils (Table 6, entry 3) [139].

In a similar study conducted by Tang et al. in 2024, comparable results were obtained. It was determined that GO exhibited reduced toxicity at lower concentrations. At concentrations exceeding 10 mg/L, a considerable number of GO particles were observed to adhere to the surface of cells, destroying cell structure and physiological function. Cadmium was also observed to exert a toxic effect on *M. aeruginosa*, resulting in cell death (EC50 of 0.51 ± 0.01 mg/L). In examining the toxic properties of GO in conjunction with cadmium, it was determined that at constant concentrations of GO, the occurrence of cell death was contingent upon the concentration of cadmium. Conversely, at constant concentrations of cadmium, the extent of cell death was dependent on the concentration of GO. The alterations in the levels of reactive oxygen species and malondialdehyde in algal cells suggest that the toxic mechanism of the GO/cadmium combination is primarily based on oxidative changes in chlorophyll and photosynthesis (Table 6, entry 4) [140].

The potential ecotoxicological risks associated with GO and the heavy metal cadmium, as well as the underlying mechanisms of toxicity on the cyanobacterium *Microcystis aeruginosa*, were investigated by Cruces et al. In this study, cyanobacteria were subjected to exposure to GO and cadmium for 96 h. The investigation of growth and photosynthesis indices revealed that concentrations exceeding 5 μg/mL of GO and 0.1 μg/mL of cadmium resulted in a decline in cell biomass and photosynthetic parameters. Additionally, GO at concentrations exceeding 9.1 μg/mL resulted in a notable decline in photochemical processes and cellular metabolism, accompanied by an elevated level of oxidative stress in *M. aeruginosa*. In conclusion, the study emphasized the necessity of considering the biological risks of GO to aquatic animals when utilizing it for the absorption of heavy metals from aquatic environments (Table 6, entry 5) [141].

In a study published in 2023, Wu et al. investigated the impact of GO on cadmium adsorption and its ecological effects on the algae *Chlorella vulgaris*. The findings indicated that GO exhibits a high adsorption capacity (120.6 mg/g) and a robust adsorption affinity (KL = 0.85 ± 0.13 L/mg) for cadmium. Additionally, the study demonstrated that cadmium is adsorbed on the surface of GO nanoparticles via cation–π interactions. The combined ecological effects of GO and cadmium on *Chlorella vulgaris* demonstrated that cadmium induced significant growth inhibition, photosynthetic toxicity, cellular structure damage, and plasmolysis when compared to GO. Furthermore, the study indicated that GO nanoparticles not only adsorbed and removed cadmium from the aquatic environment but also enhanced the toxicity of cadmium to algae. It regulates the expression of genes related to metal transport, including ABCG37 and ZIP4, and inhibits metabolic pathways, such as those involved in amino acid, fatty acid, and carbohydrate metabolism. In light of these findings, this study identifies the potential risks associated with the co‐occurrence of mineral pollutants and GO in aquatic ecosystems (Table 6, entry 6) [142].

In the study conducted by You et al., it was demonstrated that the administration of GO (400 mg L^−1^) resulted in a reduction in the expression of cadmium metal transporters, including OsIRT1, OsIRT2, OsNramp1, OsNramp5, and OsHMA2, in rice seedlings subjected to cadmium stress (by 56%–96%). Consequently, the application of GO (400 mg·L^−1^) resulted in a reduction of approximately 60% in cadmium levels in rice seedlings relative to the control condition. Nevertheless, an examination of biomass and chlorophyll fluorescence parameters indicated that GO exerted deleterious effects on plant vigor in the presence of cadmium contamination. In conclusion, although GO was observed to reduce cadmium accumulation in rice seedlings, it nevertheless exerted a negative impact on plant growth. Accordingly, a more comprehensive assessment of the dual impact of GO on crop yields is warranted [144].

In a related investigation, it was similarly determined that the impact of GO on cadmium tolerance in plants was concentration‐dependent. At low concentrations, GO has been observed to promote the growth of *Corchorus olitorius* L. and stimulate the activity of antioxidant enzymes, thereby reducing oxidative stress and improving cadmium tolerance. Nevertheless, at elevated concentrations, GO has been observed to impede plant growth and augment oxidative stress (Table 6, entry 7) [143].

Ecotoxicity findings indicate both remediation benefits and possible ecological risks, depending on the form, dose, surface chemistry, and matrix of nanoparticles, as well as the organism and exposure medium. It is evident from the preceding studies that, despite the effectiveness of graphene nanoparticles in removing the heavy metal cadmium from the environment and enhancing ecosystem conditions for living organisms, further research is required to ascertain the long‐term toxic properties of graphene nanoparticles in the environment. Recent studies have shown that using high concentrations of graphene nanoparticles for extended periods can lead to plant toxicity and hinder growth during oxidative reactions. Consequently, the exclusive consideration of the beneficial properties of nanoparticles cannot guarantee their definitive utilization in the context of bioremediation.

## 6. Discussion and Limitations

According to the evidence reviewed in the present study, this section will synthesize cross‐system insights on graphene‐based cadmium remediation, compare these approaches with established remediation strategies, and critically evaluate the robustness and generalizability of the data. Across soils, waters, plants, and biological models, a dual potential for remediation and ecological risks has been observed. Benefits of functionalized graphene materials arise from: (1) surface adsorption/complexation that reduces cadmium bioavailability and (2) modulation of metal transport, detoxification, and antioxidant pathways. Graphene form (GO vs. rGO and functionalization), dose, matrix, and exposure conditions affect the outcomes. However, it is essential to note that publication bias favoring positive results, as well as the limitations of many experiments, including synthetic or simplified matrices, small sample sizes, and short‐term assays, decrease confidence regarding viability and safety. Furthermore, long‐term environmental fate (aggregation, oxidation, interactions with natural organic matter, photochemical aging, biodegradation, and mineralization) remains uncertain and central to the durability of remediation under real‐world conditions. Chronic, field‐scale data are scarce.

Compared with activated carbon, clays, and phytoremediation without nanomaterials, graphene‐based adsorbents deliver high, tunable adsorption but raise concerns about cost, scalability, and safety. In numerous contexts, they are most effective as complementary tools rather than as universal replacements. Additionally, as graphene itself may function as both a remediation agent and a potential contaminant, it prompts regulatory and risk‐management questions about release, persistence, and nontarget effects. Explicit consideration of end‐of‐life and safe‐by‐design deployment is necessary. Overall, graphene‐based remediation exhibits potential under specific conditions. However, strong, transparent, field‐relevant studies are necessary for widespread adoption. To move forward, future studies should prioritize: (1) field‐scale, long‐term investigations with diverse matrices and multi‐contaminant conditions; (2) noncancerous biological models and a broader set of endpoints; (3) standardization of graphene materials and reporting practices; (4) preregistration and open data sharing; (5) life‐cycle risk assessments in line with regulatory standards; and (6) context‐specific, risk‐benefit deployment analyses.

## 7. Conclusion

Graphene‐derived compounds have demonstrated considerable potential for mitigating cadmium toxicity in various environmental media, including soil, wastewater, plants, food products, and biological systems. Numerous studies indicate that functionalized graphene nanomaterials can adsorb cadmium efficiently and, in some cases, mitigate its physiological effects. Many graphene‐based materials show high adsorption capacities under laboratory conditions, but their effects on biological systems are context‐dependent. Notably, GO can enhance nutrient uptake while reducing cadmium stress in plants, signaling its potential as an eco‐friendly soil amendment. Furthermore, modified graphene nanosheets showcase selective filtering capabilities in wastewater management. The application of graphene also extends to food safety, where functionalized GO nanosheets effectively eliminate cadmium from various food products. In addition, studies involving human cells indicate that graphene may act as a protective agent against cadmium’s cytotoxic effects, suggesting possible therapeutic applications.

Despite these advantages, several uncertainties persist. Most current evidence comes from short‐term laboratory experiments that may not fully reflect complex environmental conditions. In some cases, graphene derivatives may increase cadmium uptake or interact with soil and aquatic biota in unpredictable ways. Long‐term fate, transformation, bioaccumulation, and chronic low‐dose ecotoxicity of graphene‐based materials remain insufficiently understood. Therefore, while their benefits are promising, their application should proceed cautiously, with careful evaluation of ecological impacts, biological responses, and material‐specific behavior.

Future research should prioritize field‐scale validation in contaminated soils and waters, long‐term mesocosm studies, chronic ecotoxicity experiments at low and environmentally relevant concentrations, and life‐cycle assessments to clarify environmental persistence and safety. Overall, graphene‐based nanomaterials can be considered promising candidates for cadmium remediation when appropriately functionalized and applied under controlled conditions; however, they should not yet be viewed as universally superior or risk‐free alternatives, and their safe deployment requires careful evaluation of both environmental context and material‐specific behavior.

## Author Contributions

Sadaf Sadat Rafati conducted the literature search and collection, and was primarily responsible for writing the manuscript.

Elham Einafshar conceived the idea for the review article, designed the study strategy, and contributed to revisions and edits of the manuscript.

Alireza Ghassemi Toussi provided additional revisions and edits to enhance the manuscript.

## Funding

The authors declare that no funds, grants, or other support were received during the preparation of this manuscript.

## Disclosure

All authors reviewed and approved the final version.

## Ethics Statement

The authors have nothing to report.

## Consent

The authors have nothing to report.

## Conflicts of Interest

The authors declare no conflicts of interest.

## Data Availability

Data sharing is not applicable to this article as no datasets were generated or analyzed during the current study.
